# Supplementary material for: COVID-19 vaccine hesitancy among marginalized populations in the U.S. and Canada: Protocol for a scoping review
Source: PLoS One. 2022 Mar 31;17(3):e0266120. doi: 10.1371/journal.pone.0266120 (PMC8970476; doi:10.1371/journal.pone.0266120)
Supplement: S2 Appendix — (DOCX) [file pone.0266120.s002.docx]

**S2 Appendix: Sample search string**

**Medline and Embase:**

(((("corona virus" or coronavirus or COVID or nCoV) adj3 ("19" or "2019" or novel or new)) or "corona virus19" or "corona virus2019" or coronavirus19 or coronavirus2019 or COVID19 or COVID2019 or nCOV19 or nCOV2019 or "SARS Corona virus 2" or "SARS Coronavirus 2" or "SARS-COV-2" or "Severe Acute Respiratory Syndrome Corona virus 2" or "Severe Acute Respiratory Syndrome Coronavirus 2").ti,ab,hw,kw. and ((vaccin* or immunis* or immuniz*) adj3 (hesitanc* or mistrust or distrust or refus* or barrier* or confidence).ti,ab,hw,kw.))
